# Quality control material for plasma fibrinogen test produced from purified human fibrinogen

**DOI:** 10.1155/S1463924603000142

**Published:** 2003

**Authors:** Masahiro Okuda, Yahiro Uemura, Noriyuki Tatsumi

**Affiliations:** 1 Product Development Sysmex Corporation 1-1-2 Murotani Nishi-ku Kobe 651-2241 Japan; 2 Research and Development Division International Reagents Corporation 1-1-2 Murotani Nishi-ku Kobe 651-2241 Japan; 3 Department of Clinical and Laboratory Medicine Osaka City University Medical School Asahi-machi Abeno-ku Osaka 545-8585 Japan

## Abstract

Plasma fibrinogen measurement is a routine laboratory procedure commonly performed on automated coagulation analysers. Its determination is quantitative, not quantitative. Yet, a lack of precision has been an issue for fibrinogen measurement. A control material derived from plasma comprises many proteins, inhibitors and fatty acids, any or all of which can interfere in the fibrinogen assay. This study has attempted to develop a quality control material using purified human fibrinogen and has compared measurement precision between both purified and plasma materials. Purified fibrinogen was prepared using Cohn fraction 1 and glycine precipitation. Purified fibrinogen clottability was greater than 95%, with no main plasma proteins, lipids or fibrinogen degradation products observed. Two purified control materials were lyophilized at normal (2.30 g l^m1^) and abnormal (1.20 g l^m1^) levels of fibrinogen concentration. Precision was evaluated using a liquid-type reagent, Thrombocheck Fib(L), on automated coagulation analysers. Coefficient of variation for within-run, intraday and between-day precision of the purified materials was 0.7-3.5%. In comparison, the coefficient of variation for plasma materials ranged from 1.2 to 5.3%. These results suggest that materials prepared from purified fibrinogen can be useful to laboratory quality control by improving overall precision of fibrinogen measurement and are applicable to automated coagulation analysers.


**Journal of Automated Methods & Management in Chemistry**
**Vol. 25, No. 4 (July–August 2003) pp. 79–85**

### Quality control material for plasma fibrinogen test produced from purified human fibrinogen

**Masahiro Okuda†*, Yahiro Uemura‡ and Noriyuki Tatsumi§**

*†Product Development, Sysmex Corporation, 1-1-2 Murotani, Nishi-ku, Kobe 651-2241, Japan*
*‡Research and Development Division, International Reagents Corporation, 1-1-2 Murotani, Nishi-ku, Kobe 651-2241, Japan*
*§Department of Clinical and Laboratory Medicine, Osaka City University Medical School, Asahi-machi, Abeno-ku, Osaka 545-8585, Japan*

*Plasma fibrinogen measurement is a routine laboratory procedure commonly performed on automated coagulation analysers. Its determination is quantitative, not quantitative. Yet, a lack of precision has been an issue for fibrinogen measurement. A control material derived from plasma comprises many proteins, inhibitors and fatty acids, any or all of which can interfere in the fibrinogen assay. This study has attempted to develop a quality control material using purified human fibrinogen and has compared measurement precision between both purified and plasma materials. Purified fibrinogen was prepared using Cohn fraction 1 and glycine precipitation. Purified fibrinogen clottability was greater than 95%, with no main plasma proteins, lipids or fibrinogen degradation products observed. Two purified control materials were lyophilized at normal ($2.30\,g\,l^{-1}$) and abnormal ($1.20\,g\,l^{-1}$) levels of fibrinogen concentration. Precision was evaluated using a liquid-type reagent, Thrombocheck Fib(L), on automated coagulation analysers. Coefficient of variation for within-run, intraday and between-day precision of the purified materials was 0.7–3.5%. In comparison, the coefficient of variation for plasma materials ranged from 1.2 to 5.3%. These results suggest that materials prepared from purified fibrinogen can be useful to laboratory quality control by improving overall precision of fibrinogen measurement and are applicable to automated coagulation analysers.*

### Introduction

Fibrinogen is a heterogeneous glycoprotein of molecular weight (MW) 340 000 and consisting of three pairs of polypeptide chains ($A\alpha, B\beta, \gamma$) [1]. Plasma fibrinogen measurement is obtained by a screening coagulation test and many laboratories in Japan use the Clauss method [2]. Clinical studies have shown that an increased level of plasma fibrinogen is a strong predictor of atherosclerotic disease [3–5]. The difference between fibrinogen concentrations obtained by the Clauss method and those obtained by clottability assays is significantly correlated to the high molecular weight (HMW) fibrinogen of patient samples during an acute-phase reaction [6]. A wide range of fibrinogen measurement methods have been described, including biological, immunological, gravimetric and prothrombin time-derived [2,7–9]. Some manufacturers suggest calibration against the World Health Organization-prescribed international standard [10], which was based on the Clauss method and a recommended gravimetric assay (Jacobsson). Thus, a world-wide standardization of fibrinogen measurement has already been established.

In Japanese laboratories, an annual survey conducted by the Japan Medical Association (JMA) examines test performance in haematology, chemistry and immunology and its aim is improvement of quality control. In the Japanese market, multicontrol materials for prothrombin time, activated partial thromboplastin time and fibrinogen assay are commercially available; however, as they are artificially prepared from human plasma with various concentrations of fibrinogen, they are not useful for fibrinogen measurement. This is reflected by the poor record of accuracy and precision according to surveys conducted during the past 10 years [11]. Other current problems concerning fibrinogen measurement have been reported in various surveys [12]. Recently, the Hematology Subcommittee of the Standardization Committee of the Japan Society of Laboratory Medicine (JSLM) has documented a standardized method of fibrinogen measurement in plasma and established a reliable fibrinogen standard for both the Clauss method and turbidimetric immunoassay [13,14]. However, a material that produces precision and is suitable to quality control has yet to be proposed. A study was therefore undertaken to develop such a quality control material, having homogeneous composition, with no turbidity and reference material-like characteristics, in order to improve overall precision of plasma fibrinogen measurement.

The objective was to compare precision of purified materials and plasma materials for plasma fibrinogen tests using the Clauss method and to propose a quality control material suitable for plasma fibrinogen measurement.

### Experimental

### *Source plasma*

Plasma collected from 56 healthy donors was obtained from Alpha Therapeutic Corporation (Los Angeles, USA). Pooled plasma was stored at –80°C before preparation.

### *Plasma materials*

For normal control plasma, pooled plasma was used. Abnormal control plasma was prepared at half the

*To whom correspondence should be addressed.
e-mail: m-okuda@irc-net.co.jp concentration of pooled plasma and buffered with Owren veronal buffer. The two plasma materials were then lyophilized.

### *Purified fibrinogen materials*

Cohn fraction 1 [15] prepared from pooled human plasma was suspended in 55 mmol l$^{-1}$ citrate buffer (pH 6.4) containing 100 mmol l$^{-1}$ sodium chloride and 12 U ml$^{-1}$ unfractionated heparin in an ice bath and centrifuged. The supernatant was treated with a solvent detergent of 1% (v/v) Tween 80 and 0.3% (v/v) Tri-*n*-butyl phosphate (TNBP) (both Kishida Chemical, Osaka, Japan) for virus inactivation [16]. To the treated solution was added 1.8 mol l$^{-1}$ glycine. The solution was then centrifuged. This process was repeated twice and the final precipitates collected [17]. The precipitated fibrinogen was dissolved in 20 mmol l$^{-1}$ citrate buffer (pH 6.8) containing 100 mmol l$^{-1}$ sodium chloride and passed through a lysine Sepharose column (2 × 15 cm, 10 ml; Amersham Pharmacia Biotech, Tokyo, Japan) to remove plasminogen [18] (figure 1). Two lyophilized purified fibrinogen-produced materials of different concentration (2.30 and 1.20 g l$^{-1}$) were prepared in a matrix of 2.0% (w/v) protease-free bovine serum albumin (BSA) (Intergen Corporation, Georgia, USA) in 20 mmol l$^{-1}$ citrate buffer (pH 6.8) containing 100 mmol l$^{-1}$ sodium chloride.

### *Identification of fibrinogen*

The purity of the fibrinogen was evaluated by sodium dodecyl sulfate-polyacrylamide gel electrophoresis (SDS-PAGE) (Bio-Rad, Tokyo, Japan). Coomassie brilliant blue (CBB) was used for protein staining. For reduced condition, a sample solution was mixed with 1% (w/v) SDS solution at 1:4, then left standing for 30 min at 56°C, which was followed by the addition of 5% (w/v) 2-mercaptoethanol, then applied on 7.5% polyacrylamide gel containing 0.1% (w/v) SDS. For non-reduced condition, a sample solution was applied on 4% polyacrylamide gel containing 0.1% (w/v) SDS. Immunoblotting was carried out with rabbit antifibrinogen polyclonal antibody and a horseradish peroxidase conjugated goat anti-rabbit IgG antibody (both Dako-Japan, Kyoto, Japan). Clottablility of the purified fibrinogen was measured by the Laki method [19] using a UV-160 spectrophotometer (Shimadzu Corporation, Kyoto, Japan).

### *Fibrinogen measurement*

Thrombocheck Fib (L) (Sysmex Corporation, Kobe, Japan) [20] is a liquid-type reagent for use with the Clauss method, and was employed for fibrinogen measurement. The precision of within-run ($n = 20$), intraday ($n = 5$) and between-day ($n = 20$) measurements was examined by CA-1500 (Sysmex), Coagrex-700 and Coagrex-800 (Shimadzu) [21] automated coagulation analysers. The fibrinogen standard used in the assay was a commercial product (Fib STD; Sysmex), calibrated against the international reference standard prescribed by the WHO (98/612) [10], according to NCCLS procedures [22].

### *Comparison of precision of fibrinogen fractions in the preparation process*

Crude fractions of the Cohn I, and first, second and third precipitates were prepared from 10 random plasma samples during the purification process described above. Each fraction was completely dissolved in 20 mmol l$^{-1}$ citrate buffer (pH 6.8) containing 100 mmol l$^{-1}$ sodium chloride at 37°C and adjusted to 2.30 g l$^{-1}$ fibrinogen

**Fresh plasma**
↓
**Cohn fraction 1**
↓
**Virus inactivation at 30°C for 1hr**
**(treated with 1% v/v Tween 80 and 0.3% v/v TNBP)**
↓
**Glycine precipitation**
**(repeat 3 times)**
↓
**Removal of plasminogen**
**(lysine-Sepharose column)**
↓
**Sterile filtration**
↓
**Purified fibrinogen solution**
↓
**Lyophilisation**
**(about 2.30 g/L and 1.20 g/L)**

*Figure 1. Fibrinogen purification procedure.*

concentration using the Clauss method, and measured for reproducibility ($n = 20$) on the Coagrex-800.

*Residual contaminant analysis*

Analysis was performed for detection of contaminant plasma proteins (antithrombin, plasminogen, antiplasmin, coagulation factors), lipids (cholesterol, triglyceride, low density lipoprotein cholesterol (LDL), high density lipoprotein cholesterol (HDL)), turbidity (at 660 nm) and fibrinogen degradation products (FDP) in the purified fibrinogen solution and pooled plasma. The purified fibrinogen solution was adjusted in a concentration of 2.3 g $l^{-1}$ as a clottable protein using the Jacobsson method [8]. The following reagents (all Sysmex) were used: L-System AT III, PLG and APL; Thrombocheck Factors II, V, VII, X, VIII, IX, XI and XII for coagulation factors; T-CHO-K, TG-KL, HDLC-KL and LDL-C-KL for lipids; latex test BL-2 FDP for FDP assay. Turbidity for each fraction solution was prepared according to the Jacobson method [8] and measured at 660 nm wavelengths in a 1-cm cuvette using the UV-160 spectrophotometer.

*Effect of contaminated plasminogen*

A 100-μl aliquot of urokinase (1000 U $ml^{-1}$) (Mitsubishi Pharma Corporation, Osaka, Japan) solution was added to 5 ml 0.2% (w/v) purified fibrinogen solution, both before and after passage through the lysine-Sepharose column, and incubated at 37°C for 0, 6 or 18 h according to a modified procedure [18]. The reaction was stopped by addition of 1000 ATU $ml^{-1}$ aprotinin (Penthapharm-Japan, Tokyo, Japan) and one aliquot was submitted to SDS-PAGE analysis.

*Stability after reconstitution of materials*

Two quality control materials were lyophilized for stabilization, then reconstituted and stored at 11°C for 0, 1, 2, 4, 6, 8 or 12 h. Their stability after reconstitution was tested by measuring fibrinogen concentration on the Coagrex-800.

### Results

*Fibrinogen purity*

The clottability of the purified fibrinogen was 95% according to the Laki method [19] (table 1). The appearance of the purified fibrinogen solution was clear. Electrophoresis patterns under non-reducing conditions by CBB staining showed one broad band at 340 kDa, and those under reducing conditions showed its three subunits, Aα-, Bβ- and γ-chains (figure 2). No FDP were observed by SDS-PAGE.

*Precision comparison of fibrinogen fractions in the preparation*

The precision of within-run measurements ($n = 20$) of fibrinogen fractions obtained from the first, second and third precipitate fractions was better than that obtained from plasma or Cohn fraction 1 (figure 3).

*Table 1. Fibrinogen purity.*

| Before[a] (A) | After[a] (B) | Purity[b] (A – B)/A |
|---|---|---|
| 0.491 ± 0.003 | 0.022 ± 0.001 | 95% |

[a]Optimal density of purified fibrinogen solution at 280 nm and the supernatant fluid after removing a thrombin-induced clot.
[b]Purity expressed as percentage of clottable proteins.

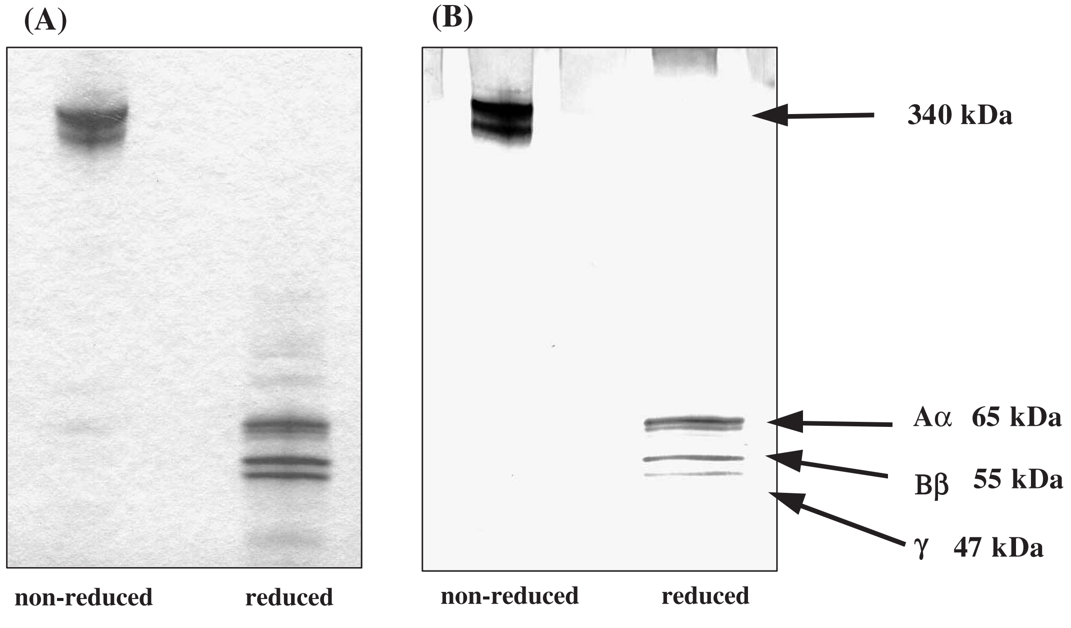

*Figure 2. SDS-PAGE patterns of purified fibrinogen. (A) CBB stain: electrophoresis, 60 V for 2.5 h; gel, 7.5% polyacrylamide; buffer, 25 mM Tris and 192 mM glycine containing 0.02% (w/v) SDS buffer (pH 7.5). (B) Western blot: membrane, PVDF membrane; buffer, 25 mM Tris and 192 mM glycine containing 20% (v/v) methanol and 0.02% (w/v) SDS buffer (pH 8.3).*

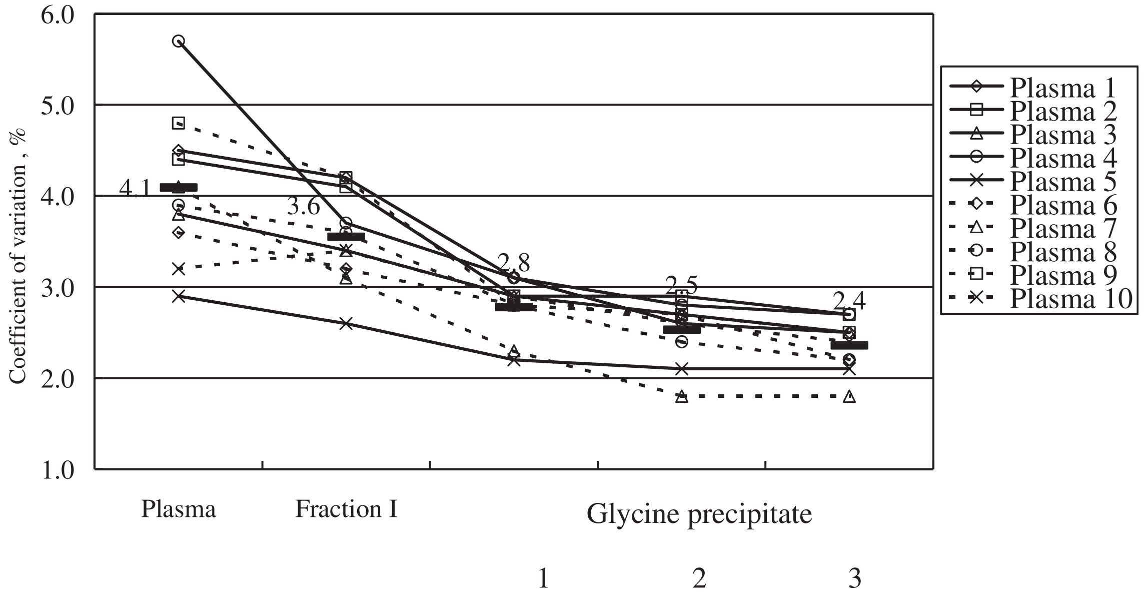

*Figure 3. Comparison of precision of fibrinogen fractions in the preparation process. The coefficient of variation (n = 20) of each fraction obtained from 10 random plasma samples was examined using Coagrex-800. Bars are the mean of CV (%).*

*Table 2. Comparison of contaminants.*

| | Preparation process | | | | |
|---|---|---|---|---|---|
| | | | Precipitate | | |
| Contaminant | Pooled plasma | Cohn fraction 1 | 1 | 2 | 3 |
| Antithrombin (%) | 103 | 8 | 6 | ND | ND |
| Plasminogen (%) | 96 | 13 | 9 | 3 | 2 |
| Antiplasmin (%) | 96 | 11 | 4 | 2 | ND |
| Intrinsic factors (%) | $95 \pm 21$ | $36 \pm 11$ | $9 \pm 3$ | ND | ND |
| Extrinsic factors (%) | $102 \pm 19$ | $11 \pm 7$ | $6 \pm 2$ | ND | ND |
| FDP ($mg\,l^{-1}$) | 3.2 | 1.2 | ND | ND | ND |
| Cholesterol ($mmol\,l^{-1}$) | 3.13 | 0.34 | 0.08 | ND | ND |
| Triglyceride ($g\,l^{-1}$) | 1.04 | 0.09 | 0.02 | ND | ND |
| LDL ($mg\,l^{-1}$) | 980 | 50 | ND | ND | ND |
| HDL ($mg\,l^{-1}$) | 660 | 50 | ND | ND | ND |
| Turbidity (660 nm) | 1.25 | 0.94 | 0.18 | 0.03 | 0.01 |

ND, not detected.

*Contaminant analysis*

Although neither plasma proteins (e.g. antithrombin, plasminogen, antiplasmin, coagulation factors, FDP) nor lipid contaminants (e.g. cholesterol, triglyceride, LDL, HDL) were observed in the purified fibrinogen material, contaminants were detected in the plasma material (table 2). The turbidity measured at 660 nm of the glycine-precipitated fibrinogen fractions was close to the detection limit (less than optical density 0.01).

*Total precision evaluation*

Coefficient of variation for within-run and between-day precision ($n = 20$) of the purified materials at normal ($2.30\,g\,l^{-1}$) and abnormal ($1.20\,g\,l^{-1}$) levels was 1.7–3.5%, while that for plasma materials was 3.1–5.3% (tables 3 and 4). Intraday ($n = 5$) CVs for purified materials and plasma materials were 0.7–1.6 and 1.2–3.0%, respectively (table 5).

*Stability*

After incubation of the purified fibrinogen with human urokinase under appropriate conditions for over 18 h at 37°C, no evidence of the subunits of FDP after treatment on the lysine-Sepharose column was observed using SDS-PAGE (figure 4). The non-treated fibrinogen solution, however, was clearly degraded. After reconstitution of the lyophilized material, the materials prepared showed good stability at 11°C for up to 12 h on the Coagrex-800 (figure 5).

### Discussion

After preparing control materials derived from a plasma source and from purified fibrinogen, their measurement precision was compared using automated coagulation analysers, the CA-1500, Coagrex-700 and Coagrex-800, all of which were equipped with light-scattering optical

*Table 3. Within-run precision.*

| | Purified material | | | | Plasma material | | | |
|---|---|---|---|---|---|---|---|---|
| | Normal | | Abnormal | | Normal | | Abnormal | |
| Instrument | Mean | CV(%) | Mean | CV(%) | Mean | CV(%) | Mean | CV(%) |
| CA-1500 | 2.28 | 2.4 | 1.19 | 2.5 | 2.31 | 3.8 | 1.21 | 4.6 |
| Coagrex-700 | 2.33 | 1.7 | 1.21 | 2.4 | 2.39 | 3.1 | 1.23 | 5.0 |
| Coagrex-800 | 2.29 | 2.3 | 1.23 | 2.8 | 2.32 | 3.3 | 1.26 | 4.3 |

Data are means expressed as fibrinogen concentration ($g\,l^{-1}$) and replicated 20 times.

*Table 4. Between-day precision.*

| | Purified material | | | | Plasma material | | | |
|---|---|---|---|---|---|---|---|---|
| | Normal | | Abnormal | | Normal | | Abnormal | |
| Instrument | Mean | CV(%) | Mean | CV(%) | Mean | CV(%) | Mean | CV(%) |
| CA-1500 | 2.27 | 2.5 | 1.16 | 3.2 | 2.30 | 3.6 | 1.19 | 4.2 |
| Coagrex-700 | 2.29 | 2.2 | 1.17 | 2.9 | 2.36 | 3.2 | 1.24 | 5.3 |
| Coagrex-800 | 2.33 | 2.4 | 1.20 | 3.5 | 2.34 | 3.5 | 1.22 | 4.2 |

Data are means expressed as fibrinogen concentration ($g\,l^{-1}$) and replicated 20 times.

*Table 5. Intraday precision.*

| | Purified material | | | | Plasma material | | | |
|---|---|---|---|---|---|---|---|---|
| | Normal | | Abnormal | | Normal | | Abnormal | |
| Instrument | Mean | CV(%) | Mean | CV(%) | Mean | CV(%) | Mean | CV(%) |
| CA-1500 | 2.23 | 0.9 | 1.17 | 1.6 | 2.33 | 1.6 | 1.24 | 2.3 |
| Coagrex-700 | 2.36 | 0.7 | 1.22 | 1.0 | 2.34 | 1.2 | 1.27 | 3.0 |
| Coagrex-800 | 2.32 | 0.7 | 1.21 | 0.9 | 2.36 | 1.8 | 1.25 | 2.2 |

Data are means expressed as fibrinogen concentration ($g\,l^{-1}$) and replicated five times.

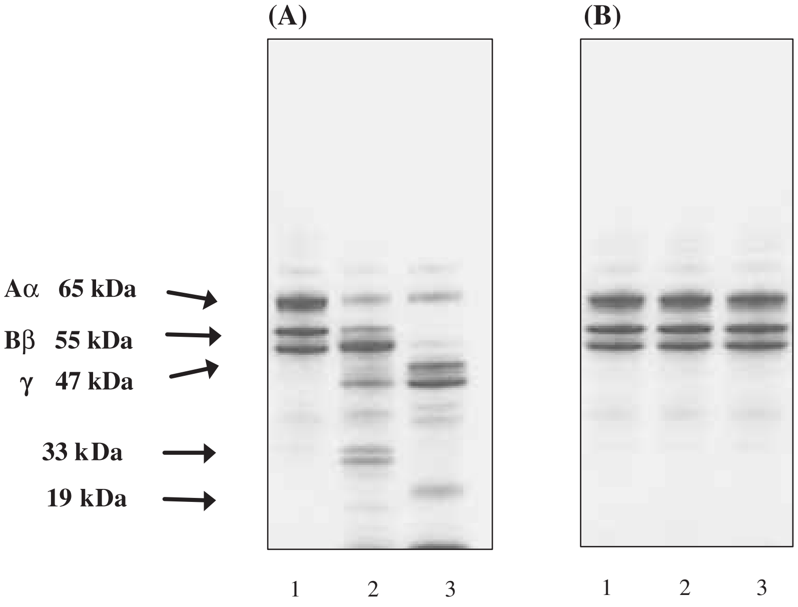

*Figure 4. SDS-PAGE of purified fibrinogen. (A) Urokinase-treated purified fibrinogen before passage through lysine-Sepharose column. (B) Urokinase-treated purified fibrinogen after passage through the column. Lanes 1, before; 2, 6 h after; 3, 18 h after incubation with urokinase. Electrophoresis, 60 V for 3.0 h; gel, 5–20% gradient polyacrylamide gel; buffer, 25 mM Tris and 192 mM glycine containing 0.02% (w/v) SDS buffer (pH 7.5); stain, CBB.*

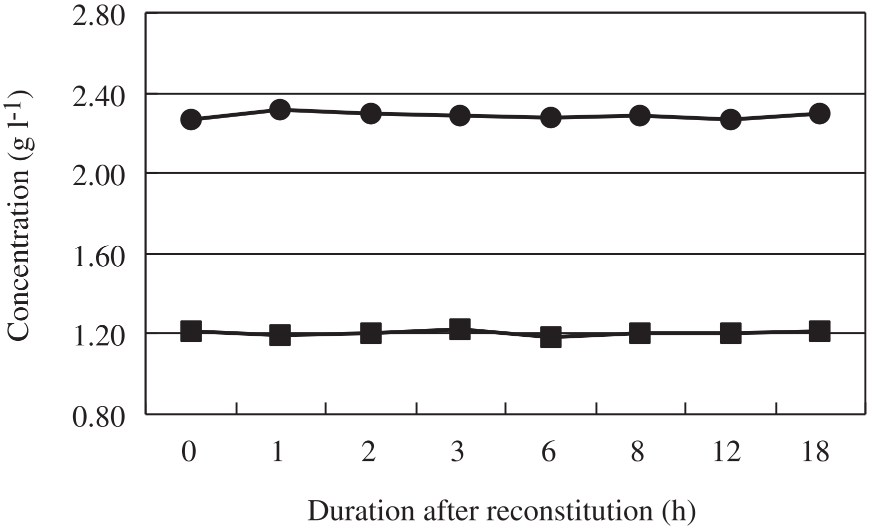

*Figure 5. Stability of purified materials after reconstitution. Fibrinogen measurement was performed using Thrombocheck Fib (L) on Coagrex-800. ●, Normal (2.2 g $l^{-1}$); ■, Abnormal (1.2 g $l^{-1}$).*

detection. The purified material showed superior precision to plasma material for all within-run, within-day and between-day measurements. Pooled plasma consists of plasma proteins (antithrombin, plasminogen, antiplasmin, coagulation factors) and lipid contaminants (such as cholesterol, triglyceride, LDL, HDL). It was assumed that these contaminants might affect the precision of fibrinogen measurement for the Clauss method. In the preparation step, the Cohn fraction 1 was yielded by ethanol precipitation, and the fibrinogen fraction was purified by glycine precipitation combining a virus-inactivated treatment by SDS denaturation and lysine-Sepharose affinity column chromatography to eliminate contaminating plasminogen. During a virus-inactivation step, detergents (Tween 80 and TNBP) can eliminate contaminants derived from lipids completely. Residual lipids and turbidity were found in the glycine-precipitate, but at lower concentrations than those found in Cohn fraction 1. However, these contaminants and turbidity were detected at much higher concentrations in pooled plasma (table 1). Thus, the purity of the purified fibrinogen was more than 95% as a clottable protein, and no other plasma proteins or lipid contaminants were observed. It was also demonstrated that the purified material solution after reconstitution was clear and its optical density at 315 nm was less than 10% of that at 280 nm according to the Jacobsson method [8]. These results suggest that turbidity and lipid or other protein contaminants may affect the precision of fibrinogen measurement; clearly, a contaminant-free material is better applied for coagulation analysers. In addition, a homogeneous reaction between fibrinogen (in the source material) and thrombin (in the reagent) may have contributed to good precision in both normal- and abnormal-level materials when using purified fibrinogen. Additionally, the safety of purified fibrinogen were confirmed when tests for HbsAg and antibodies to HIV-1, HIV-2 and HCV were all negative (data not shown).

In conclusion, the purified material rendered precision superior to that of plasma material when using the Clauss method. These characteristics make it well suited for daily quality control. Moreover, it proved free of contaminants, and was highly stable and applicable to commonly used fully automated coagulation analysers. As such, the purified material may be helpful in improving the precision of fibrinogen measurement in the clinical laboratory.

### References

1. Nieuwenhuizen, W., *Eur. Heart J.*, **16** (1995), 6.
2. Clauss, V. A., *Acta Haematol.*, **17** (1957), 237.
3. Henrich, J., Balleisn, L., Schulte, H., Assmann, G. and Van De Loo, J., *Arteriosclerosis and Thrombosis*, **14** (1994), 54.
4. Reganon, E., Vila, V., Ferrando, F., Martinez-Sales, V., Fayos, L., Ruano, M. and Aznar, J., *Thromb. Haemost.*, **82** (1999), 1403.
5. Woodward, M., Lowe, G. D., Rumley, A. and Tunstall-Pedoe, H., *Eur. Heart J.*, **19** (1998), 55.
6. Jensen, T., Halvorsen, S., Godal, H. C., Sandset, P. M. and Skjoonsberg, O. H., *Thromb. Res.*, **100** (2000), 397.
7. Hoegee-de Nobel, E., Voskuilen, M., Briet, E., Brommer, E. J. and Nieuwenhuizen, W., *Thromb. Haemost.*, **22** (1988), 4158.
8. Jacobsson, K., *Scand. J. Clin. Lab. Invest.*, **7** (1955), 1.
9. Mackie, J., Lawrie, A. S., Kitchen, S., Gaffney, P. J., Howarth, D., Lowe, G. D., Martin, J., Purdy, G., Rigsby, P. and Rumley, A., *Thromb. Haemost.*, **87** (2002), 997.
10. Whitton, C. M., Sands, D., Hubbard, A. R. and Gaffney, P. J., *Thromb. Haemost.*, **84** (2000), 258.
11. Tatsumi, N., Okuda, M., Kondo, H. and Takubo, T., *Proceedings of Asian Society for Quality Assurance in Laboratory Medicine* (2003) (in press).
12. Hirai, N., Tatsumi, N., Hino, M., Yamane, T. and Ohta, K., *Osaka City Med. J.*, **44** (1988), 55.
13. Tatsumi, N., *Rinsyou Byouri*, **49** (2001), 1273.
14. Tatsumi, N., *Rinsyou Byouri*, **49** (2001), 1280.
15. Cohn, F. J., Strong, L. E., Hughes, W. L., Jr, Mulfird, D. J., Ashworth, J. N., Melin, M. and Taylor, H. L., *J. Am. Chem. Soc.*, **68** (1946), 459.
16. Uemura, Y., Yang, Y. H., Heldebrant, C. M., Takechi, K. and Yokoyama, K. *Vox Sang*, **67** (1994), 337.
17. Okuda, M., Uemura, Y., Naka, K. and Tatsumi, N., *Clin. Lab. Hematol.*, **25** (2003), 167.

18. Matsuda, M., Iwanaga, S. and Nakamura, S. *Thromb. Res.*, **1** (1972), 619.
19. Laki, K., *Arch. Biochem. Biophys.*, **32** (1951), 317.
20. Kikukawa, N., Okuda, M., Minami, S., Ohta, Y. and Uemura, Y., *Clin. Chem.*, **47** (2001), A167.
21. Okuda, M., Kikukawa, N., Fujikawa, Y., Uemura, Y. and Yokoyama, K., *Clin. Chem.*, **46** (2000), A135.
22. National Committee for Clinical Laboratory Standardization (NCCLS). *Procedure for the Determination of Fibrinogen in Plasma: Approved Guideline*. NCCLS H30-A2 (Wayne: NCCLS, 2001).

